# Association of the Stress Hyperglycemia Ratio with Advanced Liver Fibrosis and Mortality in Patients with Metabolic Dysfunction–Associated Steatotic Liver Disease: An Analysis of NHANES

**DOI:** 10.5152/tjg.2026.25707

**Published:** 2026-02-16

**Authors:** Zhongping Su, Tao Ling, Runming Mao, Xueming Yang, Jian Wang, Yujie Xu

**Affiliations:** 1 Department of Geriatric Gastroenterology, the First Affiliated Hospital with Nanjing Medical University, Nanjing, China; 2 Department of Gastroenterology, Affiliated Jinhua Hospital, Zhejiang University School of Medicine, Jinhua, China; 3 Department of General Surgery, the Fourth Affiliated Hospital of Nanjing Medical University, Nanjing, China

**Keywords:** Advanced liver fibrosis, mortality, nonalcoholic fatty liver disease, risk, stress hyperglycemia ratio

## Abstract

**Background/Aims:**

Changes in blood glucose are correlated with metabolic dysfunction–associated steatotic liver disease (MASLD). The stress hyperglycemia ratio (SHR) serves as an innovative biomarker for accurately assessing acute hyperglycemia. This investigation aimed to analyze the correlation of SHR with advanced liver fibrosis and mortality among MASLD patients.

**Materials and Methods:**

This retrospective analysis enrolled 8078 individuals with MASLD (fatty liver index ≥60) from the National Health and Nutrition Examination Survey 1999-2018. The outcomes were advanced liver fibrosis (fibrosis 4 index >2.67), all-cause mortality (ACM), and cardiovascular disease–related mortality (CRM), and death information was recorded through December 31, 2019. The SHR was calculated using fasting plasma glucose (FPG) and glycated hemoglobin (HbA1c) according to the following formula: [FPG (mmol/L) / (1.59 × HbA1c (%) - 2.59)].

**Results:**

Among the 8078 patients, advanced liver fibrosis was identified in 243 (2.12%) patients, while ACM and CRM accounted for 1250 (9.12%) and 421 (2.92%) patients, respectively. High SHR demonstrated a correlation with higher risks of advanced liver fibrosis [odds ratio (OR, 95% CI) = 1.87 (1.15-3.04)] and ACM [hazard ratio (HR, 95% CI) = 1.45 (1.11-1.89)]. Low SHR was also related to a higher risk of ACM [HR = 1.34 (1.00-1.78)]. The SHR showed a U-shaped nonlinear relationship with advanced liver fibrosis, ACM, and CRM, and the lowest SHR values were 0.924, 0.924, and 0.945, respectively.

**Conclusion:**

The SHR exhibited a U-shaped relationship with the risk of advanced fibrosis and mortality among MASLD patients.

**Main Points**

- The stress hyperglycemic ratio (SHR) is a new indicator of acute hyperglycemia.
- The SHR showed a U-shaped relationship with advanced liver fibrosis in metabolic dysfunction–associated steatotic liver disease (MASLD) patients.
- The SHR also presented a U-shaped correlation with mortality among MASLD patients.
- The SHR may have prognostic predictive value for MASLD patients (area under the curve >0.83).

## Introduction

Metabolic dysfunction–associated steatotic liver disease (MASLD), also known as nonalcoholic fatty liver disease, has emerged as a global health burden, affecting approximately 32% of adults worldwide.^1^^,^^2^ Metabolic dysfunction–associated steatotic liver disease encompasses a continuum of hepatic pathologies, including uncomplicated steatosis, nonalcoholic steatohepatitis, progressive fibrosis, and cirrhosis.^3^ Metabolic dysfunction–associated steatotic liver disease exhibits correlation with metabolic syndromes, including obesity and type 2 diabetes, leading to increased incidence and mortality.^4^ The mortality of MASLD is primarily driven by liver-related complications (e.g., hepatocellular carcinoma) and cardiovascular diseases (CVDs),^5^ and advanced hepatic fibrosis is a major contributor to MASLD mortality.^6^ Major risk factors for MASLD include diabetes, obesity, insulin resistance, and dyslipidemia.^3^ Identifying factors correlated with the prognosis of MASLD is critical for the management of the disease.

Diabetes and poor glycemic control have been confirmed to be important factors in the progression and prognosis of MASLD.^3^^,^^7^^,^^8^ Glycated hemoglobin (HbA1c) provides a measure of mean blood glucose concentrations during the preceding 8- to 12-week period, but it cannot quantify variations in blood glucose levels among individuals. The stress hyperglycemia ratio (SHR) was developed to quantify relative hyperglycemia in acute diseases and is calculated based on fasting plasma glucose (FPG) and HbA1c.^9^ The SHR reflects the degree of stress-induced glucose fluctuations. It can capture the acute changes in fasting glucose relative to the chronic glycemic state, which also reflects the variability of blood glucose among individuals.^9^ Prolonged excessive stress hyperglycemia induces mitochondrial damage, exacerbates oxidative stress and inflammation, and leads to endothelial damage and insulin resistance, which affects the onset and prognosis of many diseases.^10^^-^^12^ Previous studies have demonstrated that impaired insulin sensitivity, sustained inflammatory responses, reactive oxygen species accumulation, and unfolded protein response activation are the main pathogenic mechanisms of MASLD.^3^^,^^13^ Several studies have found that SHR can be utilized to predict short- and long-term mortality risk in patients with diabetes, CVD, and chronic kidney disease.^10^^,^^14^^-^^17^ A recent study also found that SHR is correlated with an elevated risk of MASLD in those with diabetes or prediabetes.^18^ The presence of diabetes constitutes a relevant driver for the transition from MASLD to hepatic fibrotic progression.^19^ However, the potential impact of stress-induced glycemic changes on liver fibrosis and mortality among MASLD patients remains unsolved. Therefore, this study aimed to evaluate the connection between SHR and liver fibrosis and mortality among MASLD patients.

## Materials and Methods

### Study Design and Populations

This research employed a retrospective design with data acquisition performed through the 1999-2018 National Health and Nutrition Examination Survey (NHANES) database, specifically targeting MASLD cases. The NHANES represents a nationally representative cross-sectional assessment designed to evaluate the comprehensive health and nutritional profiles of the US population (https://www.cdc.gov/nchs/nhanes/), and the survey is administered every 2 years by the National Center for Health Statistics (NCHS). The NHANES utilizes interviews and physical examinations to collect information from participants containing demographics, nutrition-related questions, health-related questions, and laboratory tests. Metabolic dysfunction–associated steatotic liver disease patients in NHANES aged ≥20 years were initially enrolled. Exclusion criteria were applied to screen eligible patients: (1) patients with liver cancer; (2) individuals testing positive for hepatitis B surface antigen; (3) patients with positive hepatitis C antibody levels; (4) patients with heavy alcohol consumption; (5) patients with missing data on FPG; (6) patients with missing data on fasting HbA1c; (7) patients with missing data on aspartate aminotransferase (AST) or alanine aminotransferase (ALT); (8) patients with missing platelet assay data; and (9) patients with missing survival data. Participants with MASLD were identified based on a fatty liver index (FLI) ≥60.^20^^,21^ Fatty liver index was computed using the formula: FLI = e^x^ / (1 + e^x^) × 100, where x = [0.953 × ln (TG) + 0.139 × BMI + 0.718 × ln (GGT) + 0.053 × WC − 15.745] and the variables gamma-glutamyltransferase (GGT), body mass index (BMI), waist circumference (WC), and triglycerides (TG) were applied.^20^^,^^21^ The NCHS Research Ethics Review Board approved the design of NHANES. Each participant provided informed consent. This study was exempted from the review of the ethics committee and informed consent because de-identified data from a public database were used.

### Outcomes

The outcomes of the current study were advanced liver fibrosis, all-cause mortality (ACM), and CVD-related mortality (CRM). Advanced liver fibrosis was defined as a fibrosis 4 index (FIB-4) >2.67, where the FIB-4 was calculated as FIB-4 = [(age × AST) / (platelet counts × (SQRT(ALT)))].^20^ All-cause mortality encompassed death events irrespective of underlying causes. CVD-related mortality was deaths due to CVD, and CRM was identified by the relevant code in the database. Participant survival data in NHANES were sourced from the National Death Index (NDI) records (https://www.cdc.gov/nchs/data-linkage/mortality.htm). The NCHS established linkages between multiple survey datasets (including NHANES) and NDI mortality records. The Linked Mortality Files contain follow-up death records until December 31, 2019.

### Data Collection

The exposure variable of the current study was SHR. Stress hyperglycemia ratio was calculated as SHR = [FPG (mmol/L) / (1.59 × HbA1c (%) − 2.59)].^14^ In the analysis, the SHR was conducted with continuous and categorical variables [quartile (<0.849, 0.849-0.922, 0.922-1.003, ≥1.003)], respectively.

Other data of participants were collected, containing age, drinking, estimated glomerular filtration rate (eGFR), hypertension, gender, physical activity, marriage, race, smoking, dyslipidemia, education, CVD, poverty income ratio (PIR), diabetes, BMI, AST, ALT, albumin, platelet, and healthy eating index-2020 (HEI-2020) score. Heavy alcohol consumption was determined by the NHANES questionnaire (ALQ150 or ALQ151): individuals who drink 5 or more alcoholic beverages almost every day at some point in life.^22^ Physical activity was measured in terms of metabolic equivalent (MET) × exercise time (minutes), (MET·mins/week). The diagnosis of hypertension, dyslipidemia, and diabetes was established through a comprehensive assessment incorporating patient-reported medical history, pharmacological records, and confirmatory laboratory biomarkers. Cardiovascular disease was determined by the NHANES questionnaire (MCQ). The eGFR was calculated according to the already reported formula.^23^

### Statistical Analysis

The NHANES adopts a multi-stage stratified sampling design, and weighted variables from the NHANES database (SDMVPSU, WTSAF2YR, SDMVSTRA, WTSAF4YR) were applied in the analyses of this study. Measurement data were described as mean (standard error) [Mean (±S.E)], as the S.E value reflects the weighted estimate of the current sample (NHANES employs a sampling design). The comparisons between groups were made using the weighted F test. Categorical data were described as frequencies and percentages [n (%)], and comparisons between groups were made using the Rao–Scott chi-square test. Missing data in covariates (including smoking, education, marriage, and HEI-2020) were imputed using the Multiple Imputation by Chained Equations method.^24^ To enhance the robustness and stability of the interpolation, the consistency of variable distributions in 5 inferred datasets was established and evaluated. A preliminary analysis involving the calculation of means and modes for the 5 datasets was conducted to avoid overestimating the uncertainties related to the interpolation. Comparative analyses were conducted pre- and post-imputation (Supplementary Table 1).

Weighted univariable logistic regression identified confounders for advanced liver fibrosis, while weighted univariable Cox regression selected confounders for all-cause and CRM. Variables with *P* < .05 in univariable analyses were screened for confounders for inclusion in multivariable analyses by backward stepwise regression methods (Supplementary Tables 2-4). The relationships of SHR with advanced liver fibrosis, ACM, and CRM were examined. The multivariable logistic regression analysis for advanced liver fibrosis adjusted for hypertension, CVD, eGFR, and BMI, presenting results as odds ratio with 95% CI [OR (95% CI)]. Multivariable Cox regression analyses were adjusted for (1) age, gender, marriage, smoking, diabetes, CVD, eGFR, albumin, and HEI-2020 (ACM); and (2) age, gender, marriage, diabetes, CVD, and albumin (CRM); and the results were reported as hazard ratio (HR) and 95% CI. Further evaluations were conducted according to age, gender, hypertension, dyslipidemia, diabetes, and CVD subgroups. The nonlinear correlations of SHR with advanced liver fibrosis, ACM, and CRM were evaluated using restricted cubic spline (RCS). The inflection points of the RCS analysis were utilized to perform segmented multivariable analyses on both sides to explore the correlation of SHR with advanced liver fibrosis, ACM, and CRM. Kaplan–Meier curves were applied to further examine SHR-mortality relationships. Furthermore, the predictive value of SHR for advanced liver fibrosis, ACM, and CRM was assessed using the area under the curve (AUC). Statistical analyses were conducted in R 4.4.3 software (Institute for Statistics and Mathematics; Vienna, Austria), considering *P*-value <.05 as significant.

## Results

### Characteristics of the Study Population

**Figure 1.**
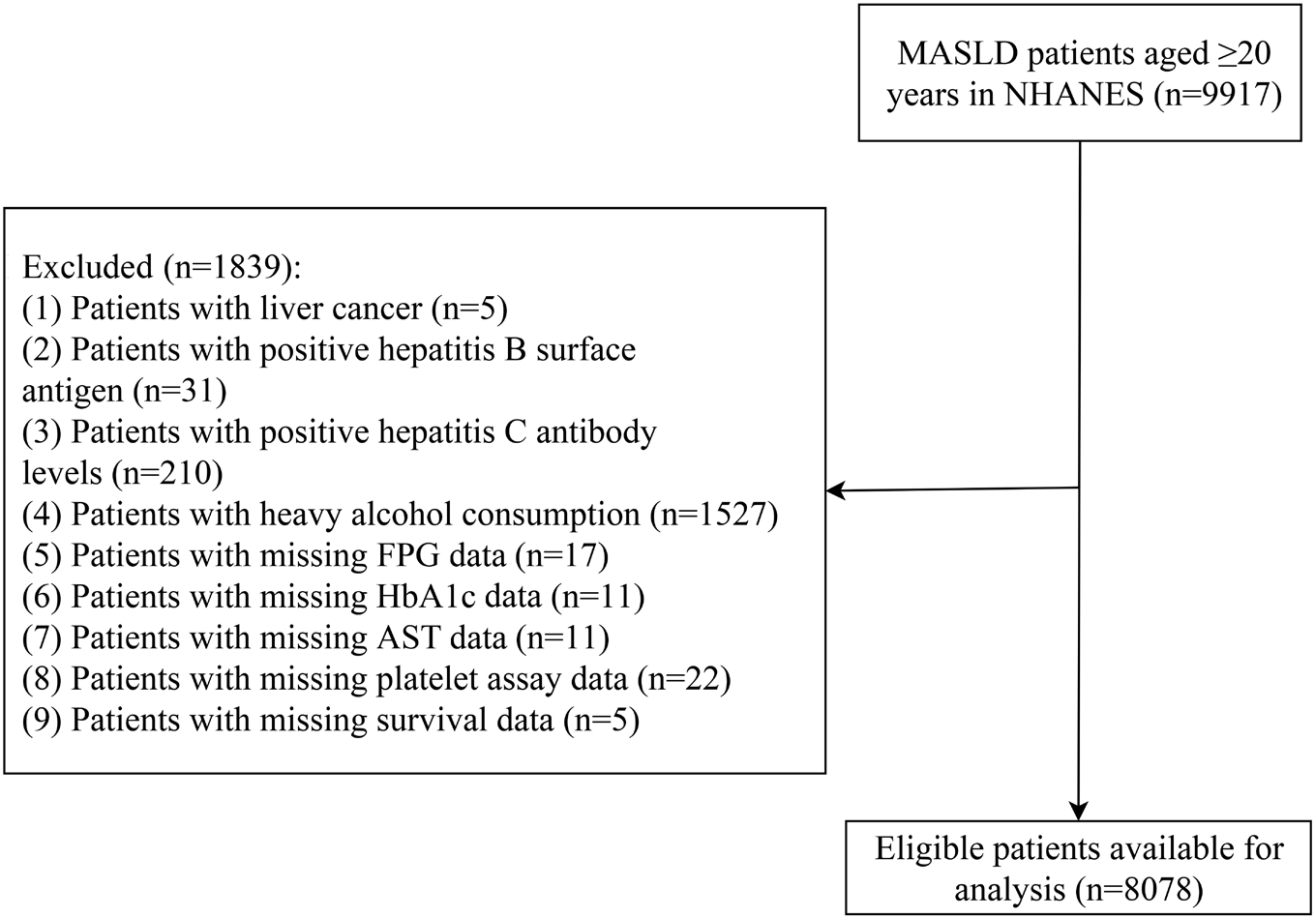
Screening flowchart for the study population. AST, aspartate aminotransferase; FBG, fasting plasma glucose; HbA1c, glycated hemoglobin; MASLD, metabolic dysfunction–associated steatotic liver disease; NHANES, the National Health and Nutrition Examination Survey.

The NHANES data from 1999 to 2018 comprised 9917 adult participants (≥20 years) diagnosed with MASLD. The screening process excluded 1839 patients, resulting in 8078 eligible patients available for analysis (Figure 1). The characteristics of 8078 MASLD patients are summarized in Table 1. The mean age of MASLD patients was 48.97 (±0.29) years, with females constituting 4169 cases (48.97%). The mean SHR value of patients was 0.94 (±0.00). Among the 8078 enrolled patients, advanced liver fibrosis was identified in 243 (2.12%) patients, while ACM and CRM accounted for 1250 (9.12%) and 421 (2.92%) patients, respectively. Significant differences emerged among patients with different SHR values in gender, race, PIR, drinking, hypertension, diabetes, eGFR, BMI, FGB, HbA1c, AST, ALT, albumin, platelet, FIB-4, advanced liver fibrosis, follow-up time, ACM, and CRM (*P* < .05).

### Relationship of Stress Hyperglycemia Ratio with Advanced Liver Fibrosis, All-Cause Mortality, and Cardiovascular Disease–Related Mortality

Elevated SHR values exhibited a significant correlation with an elevated risk of advanced liver fibrosis, both in univariable [OR (95% CI) = 7.46 (3.18-17.51)] and multivariable analyses [OR (95% CI) = 5.61 (2.71-11.63)] (Table 2). When SHR was divided into quartiles, only SHR ≥ 1.003 (Q4) [adjusted: OR (95% CI) = 1.87 (1.15-3.04)] was connected to an increased risk of advanced liver fibrosis versus 0.849-0.922 (Q2), and no correlations were found between SHR of <0.849 (Q1) and 0.922-1.003 (Q3) and advanced liver fibrosis (*P* > .05). In the analysis of mortality (Table 3), elevated SHR values were correlated with an elevated risk of ACM [adjusted: HR (95% CI) = 1.79 (1.27-2.52)] but not with the risk of CRM (adjusted: *P* = .403). Moreover, SHR of <0.849 (Q1) [adjusted: HR (95% CI) = 1.34 (1.00-1.78), *P* = .048] and ≥1.003 (Q4) [adjusted: HR (95% CI) = 1.45 (1.11-1.89)] were related to an elevated risk of ACM versus 0.849-0.922 (Q2), but only SHR of <0.849 (Q1) [adjusted: HR (95% CI) = 1.57 (1.01-2.46)] was correlated with an elevated risk of CRM.

**Figure 2.**
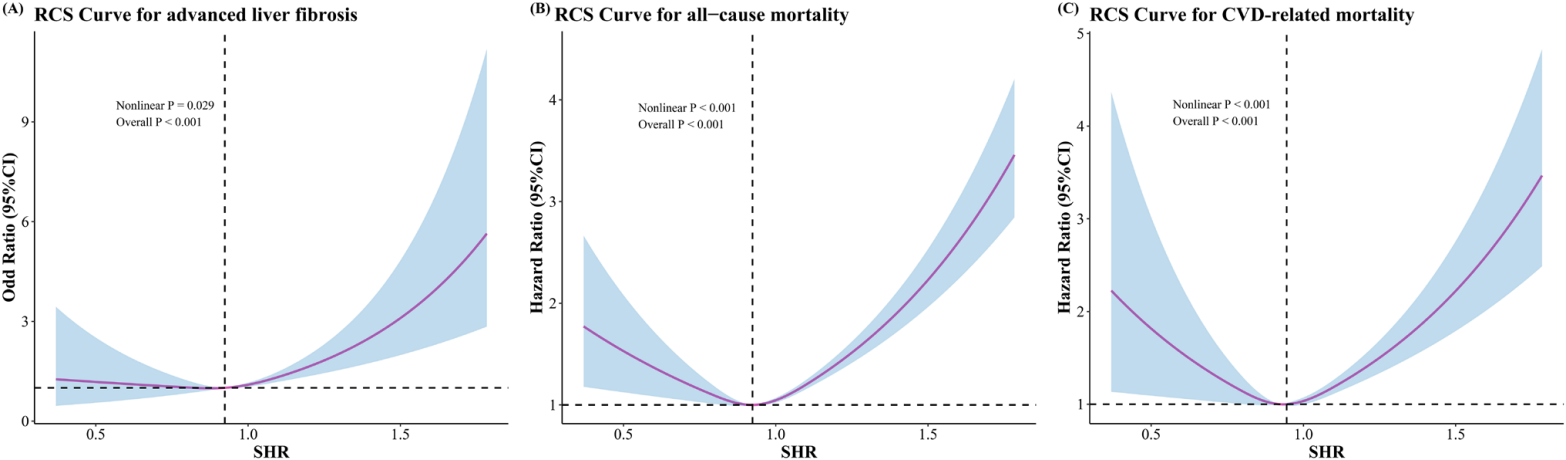
The nonlinear correlations of SHR with advanced liver fibrosis, all-cause mortality, and CVD-related mortality using restricted cubic spline (RCS). (A) advanced liver fibrosis; (B) all-cause mortality; (C) CVD-related mortality. CVD, cardiovascular diseases; SHR, stress hyperglycemia ratio.

The RCS curves demonstrated a “U” shaped nonlinear correlation around the inflection point between SHR and advanced liver fibrosis (inflection point: SHR = 0.924), ACM (SHR = 0.924), and CRM (SHR = 0.945), respectively (Figure 2). The risk of occurrence of the corresponding outcome decreased gradually before the inflection point of the SHR, and the risk of occurrence increased sharply after the inflection point of the SHR. Table 4 presents the results of the segmented multivariable analyses based on SHR inflection points. The risk of advanced liver fibrosis was increased [adjusted: OR (95% CI) = 5.27 (2.15-12.92), *P* = .048] for elevated changes in SHR above the inflection point (SHR > 0.924), and the risk of advanced liver fibrosis was not significant (*P* = .726) for elevated changes in SHR below the inflection point (SHR < 0.924). The risk of mortality increased significantly for elevated changes in SHR above the inflection point [ACM (SHR > 0.924): HR (95% CI) = 2.55 (2.00-3.24); CRM (SHR > 0.945): HR (95% CI) = 2.75 (1.40-5.39)], whereas the risk of death decreased significantly for elevated changes in SHR below the inflection point [ACM (SHR < 0.924): HR (95% CI) = 0.14 (0.05-0.38); CRM (SHR < 0.945): HR (95% CI) = 0.18 (0.05-0.67)].

**Figure 3.**
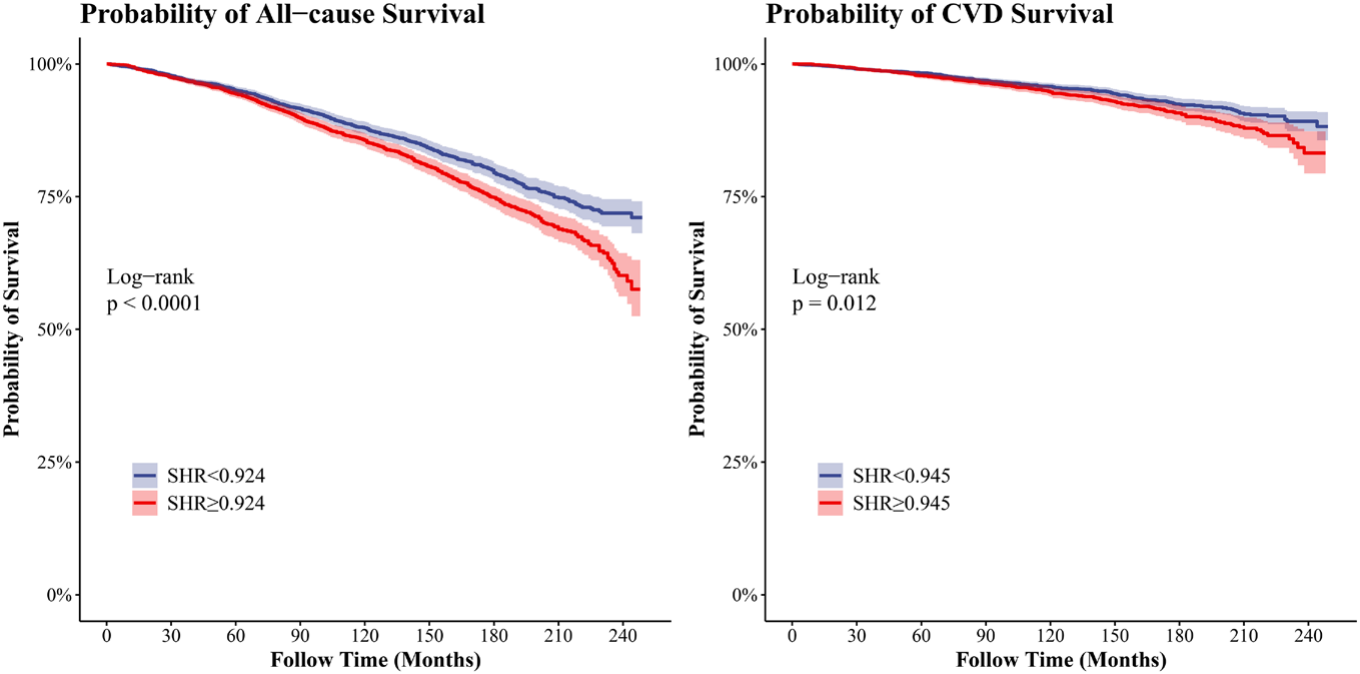
Kaplan–Meier (KM) curves for the relationship between SHR and mortality risk. (A) all-cause mortality; (B) CVD-related mortality. SHR, stress hyperglycemia ratio; CVD, cardiovascular diseases.

Kaplan–Meier curves (Figure 3) demonstrated that patients with MASLD in the SHR ≥ 0.924 group had a higher risk of ACM compared to the SHR < 0.924 group (*P* < .0001 for log-rank). Similarly, CRM risk was higher among MASLD patients with SHR values ≥0.945 compared to those below this threshold (*P* = .012 for log-rank).

**Figure 4.**
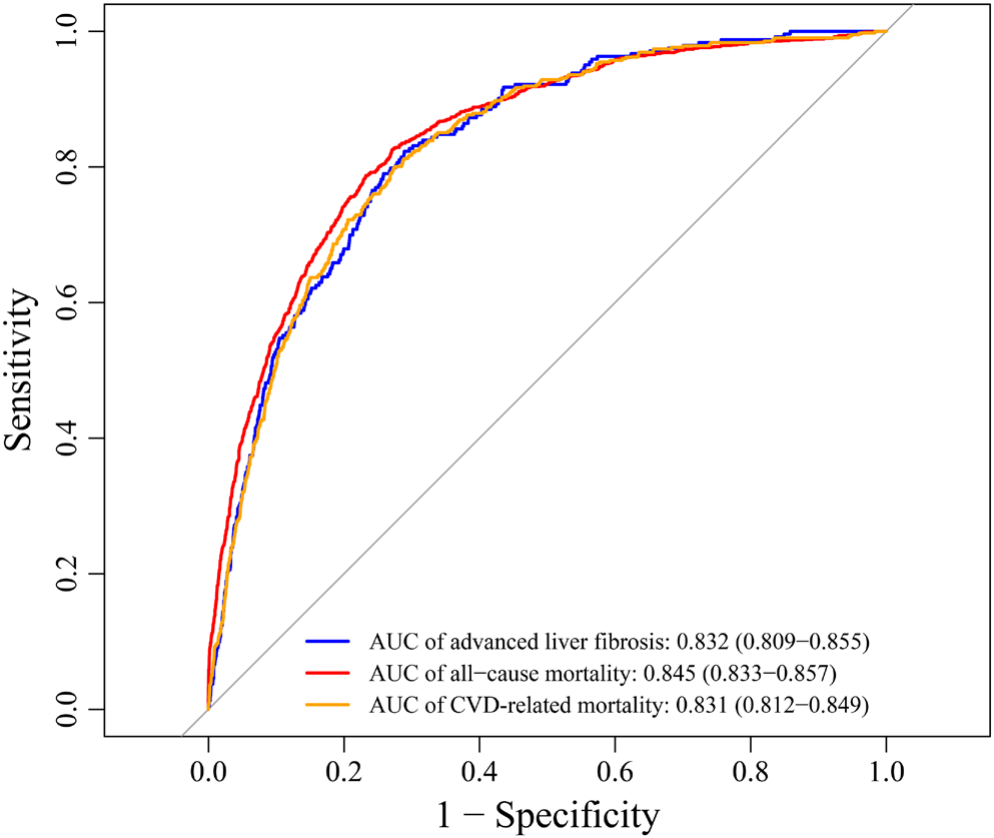
The predictive value of SHR for advanced liver fibrosis, all-cause mortality, and CVD-related mortality using the area under the curve (AUC). CVD, cardiovascular diseases; SHR, stress hyperglycemia ratio.

Furthermore, when SHR was used in combination with age, gender, and race to predict advanced liver fibrosis, ACM, and CRM, the AUCs were 0.832 (95% CI: 0.809-0.855), 0.845 (95% CI: 0.833-0.857), and 0.831 (95% CI: 0.812-0.849), respectively (Figure 4).

### Subgroup Analyses of the Correlation Between Stress Hyperglycemia Ratio and Advanced Liver Fibrosis, All-Cause Mortality, and Cardiovascular Disease–Related Mortality

**Figure 5.**
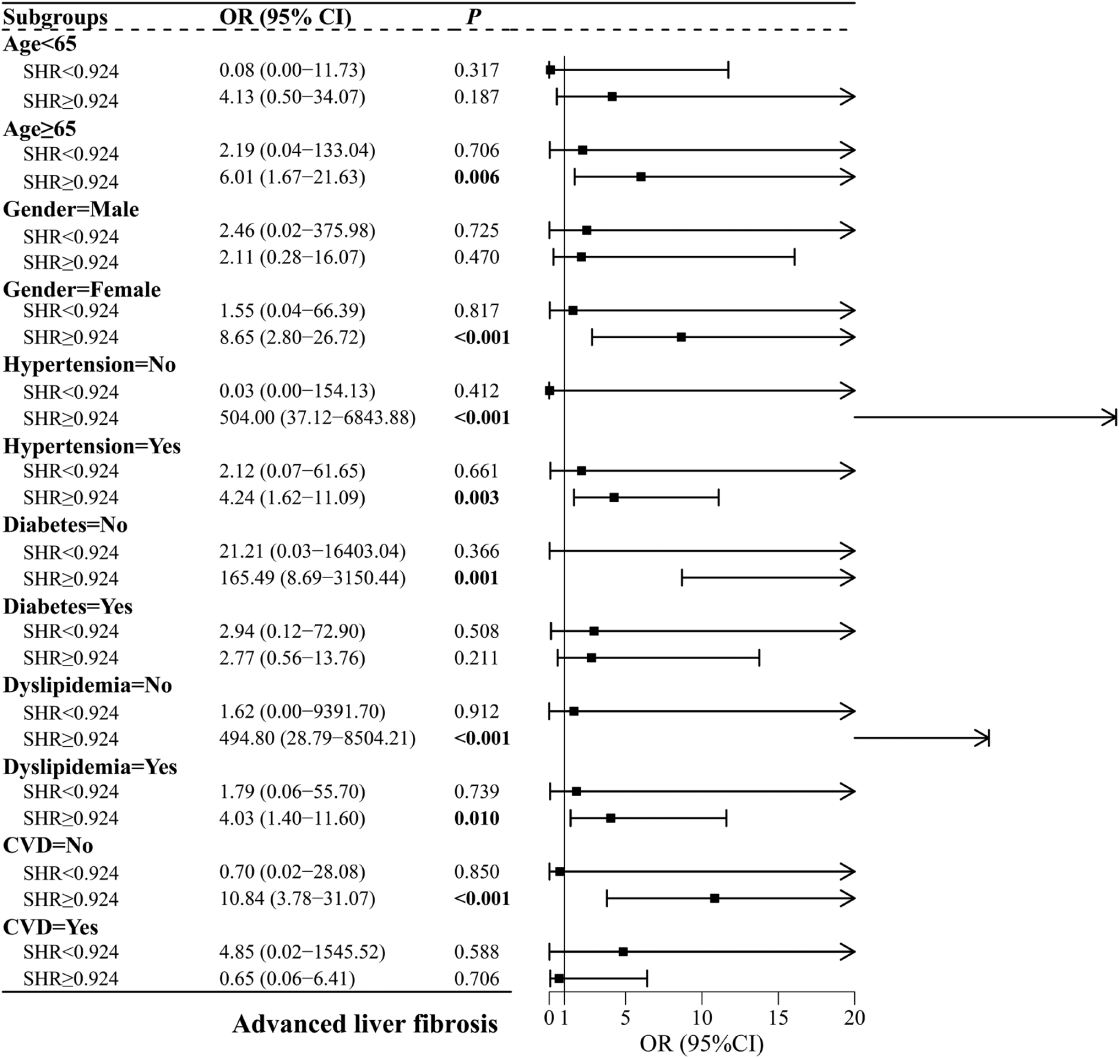
Subgroup analyses of the relationship between SHR and advanced liver fibrosis. CVD, cardiovascular diseases; SHR, stress hyperglycemia ratio.

Figure 5 presents the correlation of SHR with advanced liver fibrosis in subgroups based on the SHR inflection point. The risk of liver fibrosis was significantly increased for elevated changes in SHR > 0.924 among patients aged ≥65 years, females, those with or without hypertension, those without diabetes, dyslipidemia, and CVD (*P* < .05).

**Figure 6.**
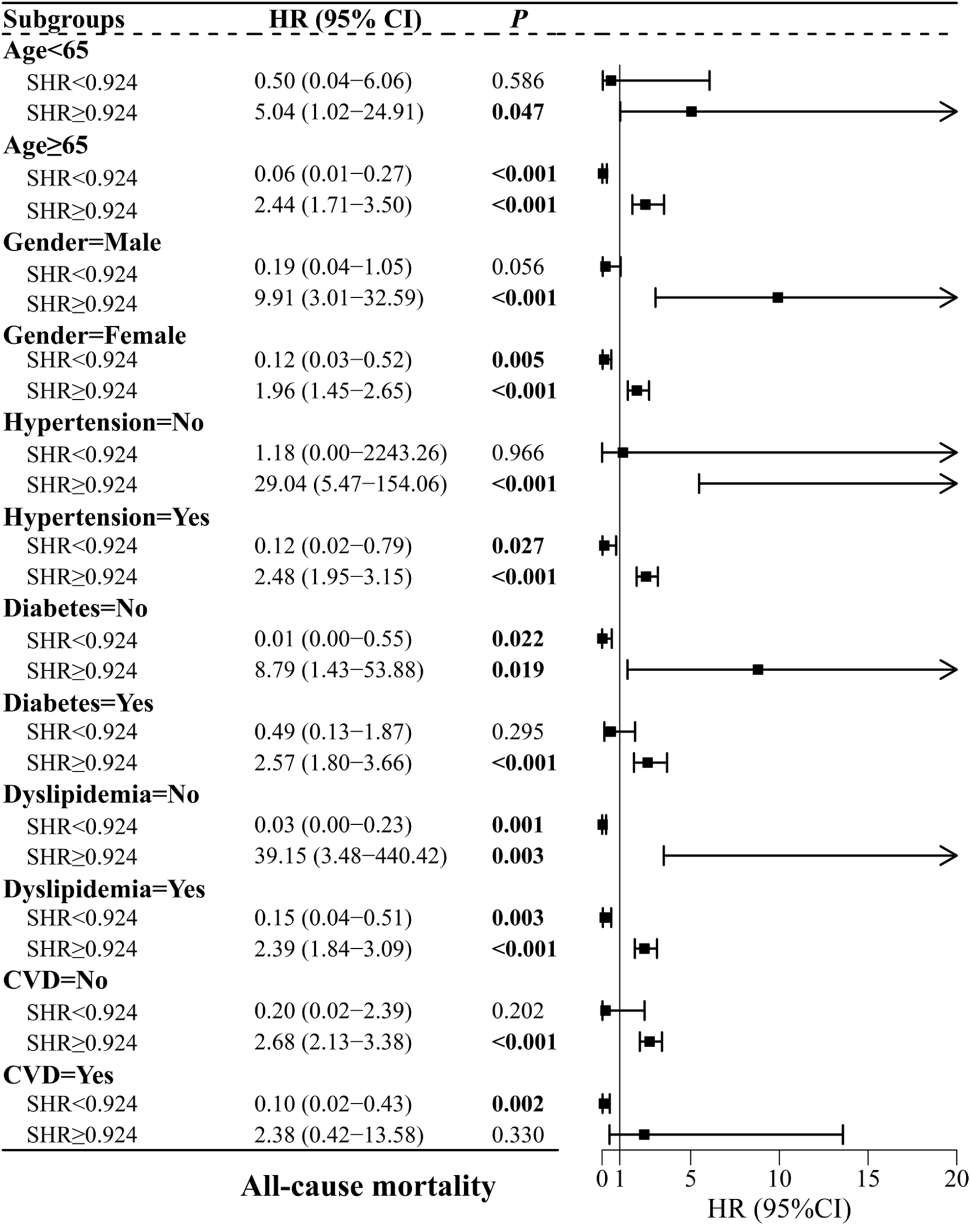
Subgroup analyses of the correlation between SHR and all-cause mortality. CVD, cardiovascular diseases; SHR, stress hyperglycemia ratio.

Figure 6 shows the correlation of SHR with ACM in subgroups based on the SHR inflection point. The risk of ACM was increased for elevated changes in SHR > 0.924 among patients aged ≥65 years or <65 years, males or females, those with or without hypertension, those with or without diabetes, those with or without dyslipidemia, and those without CVD (*P* < .05). In addition, the risk of ACM was decreased for elevated changes in SHR < 0.924 among patients aged ≥65 years, females, those with hypertension, those without diabetes, those with or without dyslipidemia, and those with CVD (*P* < .05).

**Figure 7.**
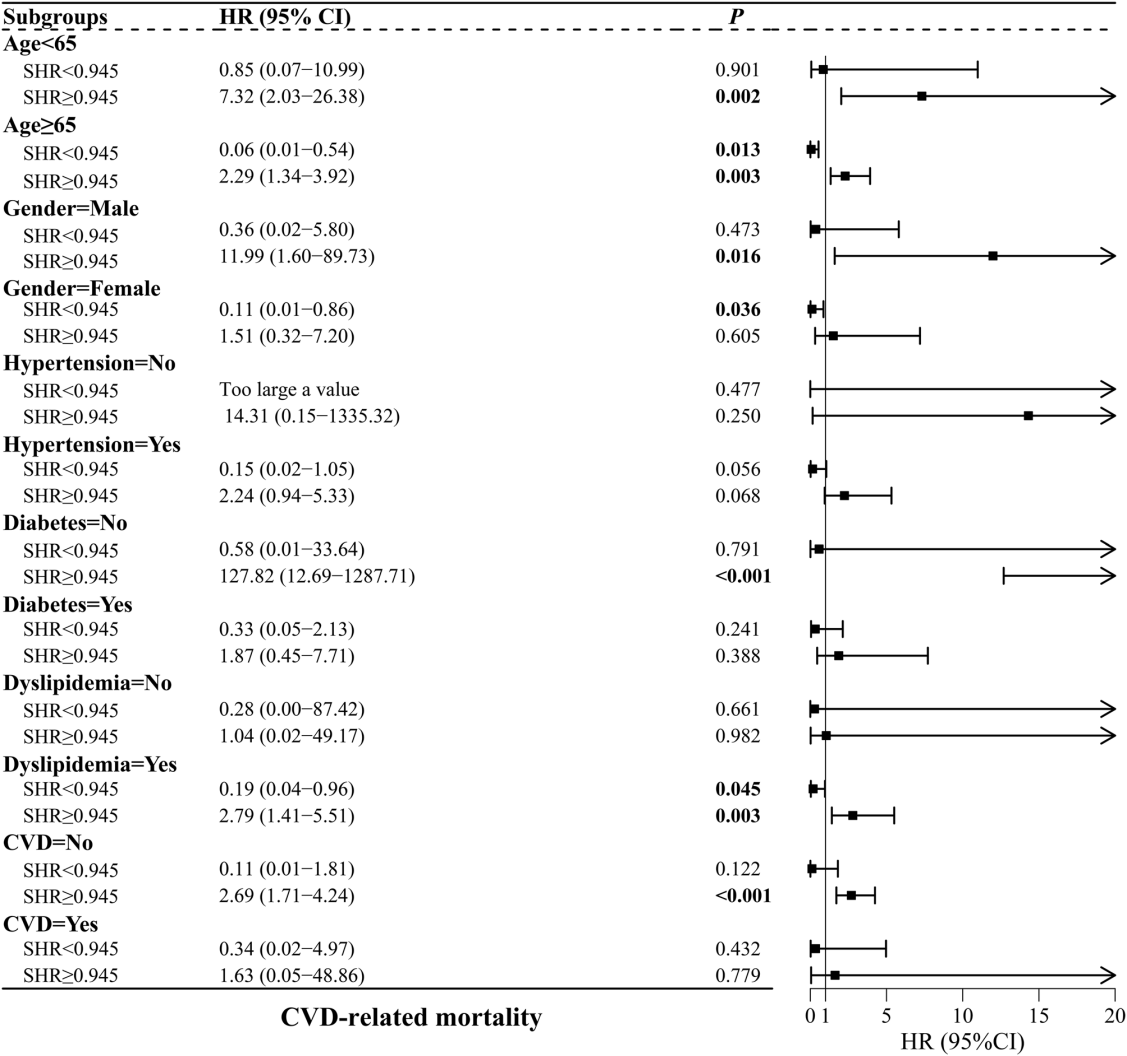
Subgroup analyses of the association between SHR and CVD-related mortality. CVD, cardiovascular diseases; SHR, stress hyperglycemia ratio.

The connections between SHR and CRM in subgroups based on the SHR inflection point are displayed in Figure 7. The risk of CRM was increased for elevated changes in SHR > 0.945 among patients aged ≥65 years or <65 years, males, individuals without diabetes, individuals with dyslipidemia, and those without CVD (*P* < .05). Furthermore, the risk of CRM was decreased for elevated changes in SHR < 0.945 among patients aged ≥65 years, females, and those with dyslipidemia (*P* < .05).

## Discussion

This analysis investigated the correlations of SHR with advanced liver fibrosis and mortality risk in MASLD patients. There was a U-shaped nonlinear relationship around the inflection point between SHR and each of the three outcomes [advanced liver fibrosis (0.924), ACM (0.924), CRM (0.945)]. Specifically, both high and low SHR were related to a higher risk of mortality. Subgroup analyses of the characteristics of different populations also revealed similar results. Furthermore, the AUC of the SHR combined with age, gender, and race for the prediction of all three outcomes exceeded 0.83.

The degree of liver fibrosis constitutes an essential biomarker for evaluating MASLD progression risk and predicting long-term clinical outcomes.^3^ Diabetes has been identified as a predictor for progression to advanced hepatic fibrosis among MASLD patients.^25^ Changes in HbA1c levels are correlated with the advancement of hepatic fibrosis among MASLD patients.^7^^,^^26^ Higher HbA1c levels are related to higher degrees of ballooned hepatocytes as well as increased liver fibrosis staging among MASLD patients.^27^ Stress hyperglycemia ratio is a good indicator to quantify the degree of stress glucose fluctuation, while chronic excessive stress hyperglycemia may induce mitochondrial damage, exacerbate oxidative stress and inflammation, lead to endothelial damage, and insulin resistance, which may affect the onset and prognosis of many diseases.^11^^,^^12^ Impaired insulin sensitivity, sustained inflammatory responses, reactive oxygen species accumulation, and unfolded protein response activation are the main pathogenic mechanisms of MASLD.^3^^,^^13^ Therefore, this investigation analyzed the relationship of SHR with advanced liver fibrosis and mortality risk among MASLD patients. These results demonstrated that SHR exhibited a “U”-shaped nonlinear relationship with advanced liver fibrosis and mortality risk. A recent study suggested that high SHR values increased the risk of MASLD, especially in patients with diabetes.^18^ This research supplements the evidence of the correlation between SHR and advanced liver fibrosis in MASLD patients. For mortality risk, previous studies have also reported a “U” shaped association between SHR and mortality risk in individuals with kidney disease,^17^ diabetes,^14^ and critical illness.^11^ The present study’s results are consistent with these findings. Moreover, it was found that SHR combined with age, gender, and race had a favorable predictive effect on advanced fibrosis and mortality risk, which provides variables for the development of subsequent risk assessment tools.

The mechanism by which SHR affects advanced liver fibrosis and mortality among MASLD patients remains unclear. The influence of high SHR on poor prognosis (advanced liver fibrosis, mortality) may be connected to the following hypotheses: insulin resistance, oxidative stress, and inflammation.^11^^,^^12^^,^^17^^,^^28^^-^^30^ Prolonged high SHR reduces insulin sensitivity, triggering excessive release of stress hormones (catecholamines, cortisol, glucagon).^31^^,^^32^ These endocrine factors contribute to elevated blood glucose concentrations, intensify oxidative stress pathways, accelerate atherosclerotic plaque formation, and induce vascular endothelial dysfunction with adverse prognostic consequences.^33^^,^^34^ Elevated SHR potentiates systemic inflammation through upregulation of key pro-inflammatory mediators, including tumor necrosis factor-α (TNF-α), interleukin-6 (IL-6), and NADPH (nicotinamide adenine dinucleotide phosphate) oxidase, thereby exacerbating damage to multiple organs.^11^ Moreover, high blood glucose levels may induce a procoagulant state, activate platelet activation and adhesion, and affect the fibrinolytic activity of fibrinolytic enzymes.^35^^-^^37^ The influence of SHR on the prognosis of patients with MASLD may also be correlated with CVD. Cardiovascular disease constitutes the predominant mortality driver among MASLD patients, and MASLD patients have a higher incidence of CVD and CRM than the general population.^38^^,^^39^ Potential mechanisms of increased CVD risk in MASLD encompass sustained inflammatory responses, compromised endothelial function, disrupted hepatic insulin signaling, accumulated oxidative damage, and metabolic lipid abnormalities.^5^

This investigation is the first to investigate the correlation of SHR with advanced liver fibrosis and mortality in MASLD patients. The analysis data are derived from the nationally representative NHANES database, which makes the findings more generalizable. However, some limitations of this study need to be considered. First, the calculation of SHR was based on the measurement results of a single FPG, which may have potential measurement errors affecting the accuracy of SHR. Second, the use of diabetes medications (e.g., insulin, oral agents) might have influenced SHR, but these factors were not accounted for. Third, SHR was initially validated in acute diseases. In chronic diseases, SHR may reflect the differences in blood glucose changes among individuals, and its application in chronic conditions requires careful interpretation. Fourth, this study employed the FIB-4 index, a composite of blood biomarkers, to assess liver fibrosis, which carries inherent limitations. Fatty liver index serves as an alternative method that may misclassify steatosis in the absence of imaging or biopsy data (e.g., transient elastography or liver biopsy). Fifth, the retrospective design resulted in findings that may have been affected by recall bias, and there were some potential confounders that were not considered. Sixth, certain liver disease–related treatment measures could not be collected and included in the analysis due to database limitations.

In conclusion, stress hyperglycemia ratio presented a “U”-shaped nonlinear correlation with advanced liver fibrosis, ACM, and CRM among MASLD patients. Both high and low SHR were correlated with an elevated risk of mortality. Moreover, SHR may have predictive value for advanced liver fibrosis and mortality.

## Supplementary Materials

Supplementary Material

## Figures and Tables

**Table 1.** Characteristics of Metabolic Dysfunction–Associated Steatotic Liver Disease Patients

**Variables**	**Total (N = 8078)**	**SHR**				***P***
		**<0.849** **(N = 2019)**	**0.849-0.922** **(N = 2020)**	**0.922-1.003** **(N = 2018)**	**≥1.003 (N = 2021)**	
Age, years, mean (±S.E.)	48.97 (±0.29)	49.61 (±0.56)	49.02 (±0.53)	48.25 (±0.50)	49.12 (±0.54)	.309
Gender, n (%)						<.001
Male	3909 (51.03)	764 (38.30)	932 (46.46)	1055 (55.12)	1158 (61.24)	
Female	4169 (48.97)	1255 (61.70)	1088 (53.54)	963 (44.88)	863 (38.76)	
Race, n (%)						<.001
Non-Hispanic White	3550 (67.46)	670 (57.50)	880 (66.02)	976 (71.54)	1024 (72.57)	
Non-Hispanic Black	1631 (11.54)	657 (21.92)	403 (11.77)	272 (6.92)	299 (7.77)	
Mexican American	1709 (10.31)	394 (10.18)	415 (10.00)	486 (11.34)	414 (9.70)	
Other Hispanic	723 (5.63)	172 (5.51)	197 (6.37)	170 (5.60)	184 (5.07)	
Other races	465 (5.05)	126 (4.88)	125 (5.84)	114 (4.59)	100 (4.88)	
Education, n (%)						.079
High school and below	4235 (42.36)	1098 (45.86)	1041 (42.86)	1025 (40.61)	1071 (40.88)	
Above high school	3843 (57.64)	921 (54.14)	979 (57.14)	993 (59.39)	950 (59.12)	
PIR, n (%)						.046
<1	1444 (12.59)	397 (15.06)	338 (11.47)	356 (12.59)	353 (11.72)	
≥1	5966 (81.52)	1441 (78.65)	1514 (81.93)	1502 (81.58)	1509 (83.31)	
Unknown	668 (5.89)	181 (6.28)	168 (6.60)	160 (5.83)	159 (4.98)	
Marriage status, n (%)						.072
Married	5164 (67.30)	1233 (63.94)	1309 (68.82)	1337 (69.01)	1285 (66.81)	
Unmarried	2914 (32.70)	786 (36.06)	711 (31.18)	681 (30.99)	736 (33.19)	
Smoking, n (%)						.904
No	4508 (56.19)	1170 (55.93)	1155 (56.86)	1117 (56.60)	1066 (55.37)	
Yes	3570 (43.81)	849 (44.07)	865 (43.14)	901 (43.40)	955 (44.63)	
Alcohol use, n (%)						.015
No	1206 (11.94)	358 (14.42)	312 (12.21)	263 (11.20)	273 (10.49)	
Yes	6332 (82.86)	1490 (78.77)	1585 (83.08)	1622 (84.07)	1635 (84.65)	
Unknown	540 (5.20)	171 (6.82)	123 (4.71)	133 (4.74)	113 (4.86)	
Physical activity, n (%)						.116
<600	1388 (17.50)	361 (19.34)	359 (18.11)	344 (15.38)	324 (17.58)	
≥600	3778 (54.36)	876 (50.62)	943 (53.43)	972 (56.24)	987 (56.30)	
Unknown	2912 (28.14)	782 (30.04)	718 (28.46)	702 (28.38)	710 (26.12)	
Hypertension, n (%)						.044
No	2656 (35.83)	658 (36.42)	713 (38.24)	699 (36.82)	586 (32.11)	
Yes	5422 (64.17)	1361 (63.58)	1307 (61.76)	1319 (63.18)	1435 (67.89)	
Diabetes, n (%)						<.001
No	5746 (75.27)	1473 (74.95)	1663 (84.64)	1569 (81.42)	1041 (60.70)	
Yes	2332 (24.73)	546 (25.05)	357 (15.36)	449 (18.58)	980 (39.30)	
Dyslipidemia, n (%)						.434
No	1243 (16.38)	339 (16.07)	335 (17.77)	305 (16.59)	264 (15.10)	
Yes	6835 (83.62)	1680 (83.93)	1685 (82.23)	1713 (83.41)	1757 (84.90)	
CVD, n (%)						.055
No	6763 (86.49)	1680 (85.28)	1744 (88.84)	1706 (86.84)	1633 (84.89)	
Yes	1315 (13.51)	339 (14.72)	276 (11.16)	312 (13.16)	388 (15.11)	
eGFR, mL/min/1.73m^2^, mean (±S.E.)	95.09 (±0.37)	93.04 (±0.66)	95.39 (±0.60)	95.96 (±0.67)	95.54 (±0.69)	.009
BMI, n (%)						.048
<30	2072 (23.06)	431 (20.20)	493 (21.81)	566 (25.44)	582 (24.14)	
≥30	6006 (76.94)	1588 (79.80)	1527 (78.19)	1452 (74.56)	1439 (75.86)	
AST, U/L, mean (±S.E.)	26.09 (±0.26)	25.44 (±0.50)	25.56 (±0.60)	25.96 (±0.54)	27.21 (±0.41)	.021
ALT, U/L, mean (±S.E.)	29.50 (±0.30)	27.04 (±0.53)	28.41 (±0.58)	29.25 (±0.55)	32.69 (±0.67)	<.001
Albumin, g/L, mean (±S.E.)	41.57 (±0.08)	40.63 (±0.12)	41.48 (±0.10)	41.97 (±0.12)	42.01 (±0.14)	<.001
Platelet, K/uL, mean (±S.E.)	254.55 (±1.16)	265.67 (±2.44)	259.43 (±1.98)	254.18 (±2.07)	241.67 (±2.20)	<.001
HEI-2020, score, mean (±S.E.)	50.92 (±0.21)	51.03 (±0.37)	51.36 (±0.39)	50.31 (±0.42)	51.02 (±0.41)	.241
SHR, mean (±S.E.)	0.94 (±0.00)	0.79 (±0.00)	0.89 (±0.00)	0.96 (±0.00)	1.10 (±0.00)	<.001
FBG, mmol/L, mean (±S.E.)	6.30 (±0.03)	5.48 (±0.03)	5.80 (±0.03)	6.16 (±0.03)	7.54 (±0.09)	<.001
HbA1c, %, mean (±S.E.)	5.83 (±0.02)	6.06 (±0.03)	5.74 (±0.02)	5.66 (±0.02)	5.90 (±0.04)	<.001
FIB-4, mean (±S.E.)	1.05 (±0.01)	1.02 (±0.02)	1.00 (±0.02)	1.03 (±0.02)	1.12 (±0.03)	.002
Advanced liver fibrosis, n (%)						.008
No	7835 (97.88)	1972 (98.36)	1967 (98.38)	1960 (98.18)	1936 (96.77)	
Yes	243 (2.12)	47 (1.64)	53 (1.62)	58 (1.82)	85 (3.23)	
Follow-up time in months (±S.E.)	101.63 (±1.50)	104.67 (±1.96)	102.86 (±2.32)	105.20 (±2.31)	94.63 (±2.17)	<.001
All-cause mortality, n (%)						.003
Survival	6828 (90.88)	1702 (89.21)	1770 (92.84)	1720 (91.65)	1636 (89.56)	
Death	1250 (9.12)	317 (10.79)	250 (7.16)	298 (8.35)	385 (10.44)	
CVD-related mortality, n (%)						.007
Survival	6828 (90.88)	1702 (89.21)	1770 (92.84)	1720 (91.65)	1636 (89.56)	
Death of CVD	421 (2.92)	118 (4.07)	77 (2.24)	95 (2.41)	131 (3.17)	
Death of other causes	829 (6.20)	199 (6.72)	173 (4.92)	203 (5.94)	254 (7.27)	

ALT, alanine aminotransferase; AST, aspartate aminotransferase; BMI, body mass index; CVD, cardiovascular diseases; eGFR, estimated glomerular filtration rate; FBG, fasting plasma glucose; FIB-4, fibrosis 4 index.; HbA1c, glycated hemoglobin; HEI-2020, healthy eating index-2020; MASLD, metabolic dysfunction–associated steatotic liver disease; PIR, poverty income ratio; SHR, stress hyperglycemia ratio (quartile: <0.849, 0.849-0.922, 0.922-1.003, ≥1.003).

**Table 2.** The Relationship Between Stress Hyperglycemic Ratio and Advanced Liver Fibrosis

**Variables**	**Model 1**		**Model 2**	
	**OR (95% CI)**	***P***	**OR (95% CI)**	***P***
SHR	7.46 (3.18-17.51)	<.001	5.61 (2.71-11.63)	<.001
SHR				
0.849-0.922	Ref		Ref	
<0.849	1.01 (0.54-1.91)	.969	0.85 (0.44-1.64)	.622
0.922-1.003	1.13 (0.73-1.75)	.590	1.12 (0.71-1.77)	.617
≥1.003	2.03 (1.24-3.32)	.005	1.87 (1.15-3.04)	.012

OR, odds ratio; Ref, reference; SHR, stress hyperglycemia ratio.

Model 1 is a univariable logistic regression analysis.

Model 2 is a multivariable logistic regression analysis adjusted for hypertension, cardiovascular disease, estimated glomerular filtration rate, and body mass index.

**Table 3.** The Relationship of Stress Hyperglycemic Ratio with All-Cause and Cardiovascular Disease–Related Mortality

**Outcome**	**Variables**	**Model 1**		**Model 2**	
		**HR (95% CI)**	***P***	**HR (95% CI)**	***P***
All-cause mortality	SHR	2.49 (1.65-3.76)	<.001	1.79 (1.27-2.52)	.001
	SHR				
	0.849-0.922	Ref		Ref	
	<0.849	1.50 (1.13-1.98)	.004	1.34 (1.00-1.78)	.048
	0.922-1.003	1.13 (0.86-1.49)	.372	1.18 (0.90-1.57)	.236
	≥1.003	1.64 (1.27-2.11)	<.001	1.45 (1.11-1.89)	.006
CVD-related mortality	SHR	0.77 (0.17-3.57)	.741	0.64 (0.22-1.83)	.403
	SHR				
	0.849-0.922	Ref		Ref	
	<0.849	1.80 (1.20-2.70)	.004	1.57 (1.01-2.46)	.045
	0.922-1.003	1.05 (0.65-1.70)	.848	1.05 (0.67-1.67)	.821
	≥1.003	1.59 (1.05-2.40)	.028	1.29 (0.83-2.01)	.252

CVD, cardiovascular disease; HR, hazard ratio; Ref, reference; SHR, stress hyperglycemia ratio.

Model 1 is a univariable Cox regression analysis.

Model 2 is a multivariable Cox regression analysis adjusted for (1) age, gender, marriage, smoking, diabetes, CVD, estimated glomerular filtration rate, albumin, and HEI-2020 (all-cause mortality); and (2) age, gender, marriage, diabetes, CVD, and albumin (CVD-related mortality).

**Table 4.** The Segmented Multivariable Analyses Based on Stress Hyperglycemia Ratio Inflection Points

**Outcomes**	**Variables**	**OR (95% CI)**	***P***
Advanced liver fibrosis	SHR inflection points	0.924	
	SHR < 0.924	1.74 (0.08-39.02)	.726
	SHR ≥ 0.924	5.27 (2.15-12.92)	<.001
		**HR (95% CI)**	***P***
All-cause mortality	SHR inflection points	0.924	
	SHR < 0.924	0.14 (0.05-0.38)	<.001
	SHR ≥ 0.924	2.55 (2.00-3.24)	<.001
CVD-related mortality	SHR inflection points	0.945	
	SHR < 0.945	0.18 (0.05-0.67)	.010
	SHR ≥ 0.945	2.75 (1.40-5.39)	.003

CVD, cardiovascular diseases; HR, hazard ratio; OR, odds ratio; SHR, stress hyperglycemia ratio.

Multivariable logistic regression analysis adjusted for hypertension, CVD, estimated glomerular filtration rate (eGFR), and body mass index (advanced liver fibrosis).

Multivariable Cox regression analysis adjusted for (1) age, gender, marriage, smoking, diabetes, CVD, eGFR, albumin, and HEI-2020 (all-cause mortality) and (2) age, gender, marriage, diabetes, CVD, and albumin (CVD-related mortality).

## Data Availability

The data that support the findings of this study are available on request from the corresponding author.
