# Supplementary material for: Association of the Stress Hyperglycemia Ratio with Advanced Liver Fibrosis and Mortality in Patients with Metabolic Dysfunction–Associated Steatotic Liver Disease: An Analysis of NHANES
Source: Turk J Gastroenterol. 2026 Feb 16;37(4):483–96. doi: 10.5152/tjg.2026.25707 (PMC13047330; doi:10.5152/tjg.2026.25707)
Supplement: Supplementary Material [file supplementary_material.pdf]

Supplementary Table 1. Comparative analyses pre- and post-imputation of missing values. HEI-2020, healthy eating index-2020

Variables	Pre-imputation	Post-imputation	Statistics	*P*
Smoking, n (%)			χ^2 = 0.176	0.676
No	4506 (56.19)	4508 (56.19)		
Yes	3569 (43.81)	3570 (43.81)		
Education, n (%)			χ^2 = 3.518	0.063
High School and below	4230 (42.35)	4235 (42.36)		
Above high school	3843 (57.65)	3843 (57.64)		
Marriage, n (%)			χ^2 = 1.586	0.210
Married	5108 (67.28)	5164 (67.30)		
Unmarried	2887 (32.72)	2914 (32.70)		
HEI-2020, Mean (±S.E)	50.89 (±0.22)	50.92 (±0.21)	*t* = 1.656	0.100

Supplementary Table 2. Confounders related to advanced liver fibrosis using logistic regression analysis

	Univariable analysis		Multivariable analysis (backward stepwise regression)	
Variables	OR (95% CI)	*P*	OR (95% CI)	*P*
Age	1.10 (1.09-1.12)	<0.001		
Gender				
Male	Ref			
Female	0.88 (0.61-1.27)	0.484		
Race				
Non-Hispanic White	Ref			
Non-Hispanic Black	0.73 (0.44-1.20)	0.210		
Mexican American	0.94 (0.57-1.55)	0.808		
Other Hispanic	0.64 (0.36-1.12)	0.115		
Other Race	0.54 (0.18-1.63)	0.272		
Education				
High School and below	Ref			
Above high school	0.77 (0.52-1.14)	0.190		
PIR				
<1	Ref			
≥1	1.52 (0.85-2.71)	0.158		
Unknown	1.22 (0.53-2.81)	0.645		
Marriage				
Married	Ref			
Unmarried	1.29 (0.87-1.89)	0.199		
Smoking				
No	Ref			
Yes	1.26 (0.86-1.85)	0.233		

(*Continued*)

Supplementary Table 2. Confounders related to advanced liver fibrosis using logistic regression analysis (*Continued*)

	Univariable analysis		Multivariable analysis (backward stepwise regression)	
Variables	OR (95% CI)	*P*	OR (95% CI)	*P*
Drinking				
No	Ref			
Yes	0.70 (0.43-1.16)	0.163		
Unknown	0.97 (0.38-2.49)	0.947		
Physical activity				
<600	Ref			
≥600	0.84 (0.48-1.45)	0.522		
Unknown	1.27 (0.70-2.29)	0.434		
Hypertension				
No	Ref		Ref	
Yes	6.89 (4.24-11.19)	<0.001	3.75 (2.20-6.39)	<0.001
Diabetes				
No	Ref			
Yes	2.67 (1.86-3.84)	<0.001		
Dyslipidemia				
No	Ref			
Yes	1.03 (0.64-1.68)	0.888		
CVD				
No	Ref		Ref	
Yes	4.65 (3.14-6.90)	<0.001	2.20 (1.51-3.22)	<0.001
eGFR	0.96 (0.96-0.97)	<0.001	0.98 (0.97-0.98)	<0.001
BMI				
<30	Ref		Ref	
≥30	0.60 (0.40-0.91)	0.015	0.61 (0.40-0.94)	0.027
Albumin	0.93 (0.86-0.99)	0.026		
HEI-2020	1.02 (1.01-1.03)	0.008		

Note: PIR, poverty income ratio; CVD, cardiovascular diseases; eGFR, estimated glomerular filtration rate; BMI, body mass index; HEI-2020, healthy eating index-2020; OR, odds ratio; Ref, reference.

Supplementary Table 3. Confounders related to all-cause mortality using Cox regression analysis

	Univariable analysis		Multivariable analysis (backward stepwise regression)	
Variables	HR (95% CI)	*P*	HR (95% CI)	*P*
Age	1.10 (1.09-1.11)	<0.001	1.08 (1.07-1.10)	<0.001
Gender				
Male	Ref		Ref	
Female	0.84 (0.70-1.00)	0.048	0.54 (0.45-0.64)	<0.001
Race				
Non-Hispanic White	Ref			
Non-Hispanic Black	0.78 (0.56-1.09)	0.141		
Mexican American	0.41 (0.19-0.89)	0.024		
Other Hispanic	0.41 (0.14-1.20)	0.104		
Other Race	0.69 (0.45-1.06)	0.087		
Education				
High School and below	Ref			
Above high school	0.63 (0.54-0.73)	<0.001		
PIR				
<1	Ref			
≥1	0.97 (0.66-1.43)	0.885		
Unknown	1.11 (0.59-2.09)	0.738		
Marriage				
Married	Ref		Ref	
No married	1.33 (1.06-1.68)	0.013	1.34 (1.08-1.66)	0.007
Smoking				
No	Ref		Ref	
Yes	1.88 (1.55-2.29)	<0.001	1.29 (1.06-1.57)	0.010
Drinking				
No	Ref			
Yes	0.74 (0.59-0.93)	0.009		
Unknown	0.68 (0.33-1.41)	0.298		
Physical activity				
<600	Ref			
≥600	0.85 (0.64-1.14)	0.280		
Unknown	2.14 (1.63-2.80)	<0.001		
Hypertension				
No	Ref			
Yes	3.57 (2.51-5.07)	<0.001		
Diabetes				
No	Ref		Ref	
Yes	3.20 (2.73-3.76)	<0.001	1.40 (1.21-1.63)	<0.001

(*Continued*)

Supplementary Table 3. Confounders related to all-cause mortality using Cox regression analysis (*Continued*)

	Univariable analysis		Multivariable analysis (backward stepwise regression)	
Variables	HR (95% CI)	*P*	HR (95% CI)	*P*
Dyslipidemia				
No	Ref			
Yes	1.79 (1.20-2.68)	0.005		
CVD				
No	Ref		Ref	
Yes	6.16 (5.44-6.98)	<0.001	1.86 (1.62-2.14)	<0.001
eGFR	0.96 (0.96-0.96)	<0.001	0.99 (0.99-1.00)	0.019
BMI				
<30	Ref			
≥30	0.80 (0.65-1.00)	0.048		
Albumin	0.93 (0.91-0.95)	<0.001	0.91 (0.89-0.95)	<0.001
HEI-2020	1.01 (1.00-1.02)	0.007	0.99 (0.98-1.00)	0.003

Note: PIR, poverty income ratio; CVD, cardiovascular diseases; eGFR, estimated glomerular filtration rate; BMI, body mass index; HEI-2020, healthy eating index-2020; HR, hazard ratio; Ref, reference.

Supplementary Table 4. Confounders related to CVD-related mortality using Cox regression analysis

	Univariable analysis		Multivariable analysis (backward stepwise regression)	
Variables	HR (95% CI)	*P*	HR (95% CI)	*P*
Age	1.11 (1.09-1.13)	<0.001	1.09 (1.07-1.12)	<0.001
Gender				
Male	Ref		Ref	
Female	0.72 (0.54-0.95)	0.022	0.38 (0.28-0.52)	<0.001
Race				
Non-Hispanic White	Ref			
Non-Hispanic Black	1.19 (0.77-1.85)	0.430		
Mexican American	0.39 (0.08-1.85)	0.238		
Other Hispanic	0.57 (0.17-1.87)	0.351		
Other Race	0.60 (0.31-1.16)	0.130		
Education				
High School and below	Ref			
Above high school	0.56 (0.40-0.77)	<0.001		
PIR				
<1	Ref			
≥1	0.89 (0.53-1.47)	0.638		
Unknown	1.28 (0.51-3.19)	0.601		

(*Continued*)

Supplementary Table 4. Confounders related to CVD-related mortality using Cox regression analysis (*Continued*)

	Univariable analysis		Multivariable analysis (backward stepwise regression)	
Variables	HR (95% CI)	*P*	HR (95% CI)	*P*
Marriage				
Married	Ref		Ref	
No married	1.69 (1.23-2.31)	0.001	1.83 (1.30-2.60)	0.001
Smoking				
No	Ref			
Yes	1.70 (1.27-2.27)	<0.001		
Drinking				
No	Ref			
Yes	0.76 (0.51-1.15)	0.194		
Unknown	0.36 (0.08-1.58)	0.176		
Physical activity				
<600	Ref			
≥600	0.86 (0.56-1.33)	0.509		
Unknown	2.16 (1.40-3.35)	0.001		
Hypertension				
No	Ref			
Yes	5.17 (2.40-11.13)	<0.001		
Diabetes				
No	Ref		Ref	
Yes	3.70 (2.80-4.88)	<0.001	1.53 (1.18-2.00)	0.002
Dyslipidemia				
No	Ref			
Yes	1.99 (1.13-3.48)	0.016		
CVD				
No	Ref		Ref	
Yes	9.08 (7.21-11.43)	<0.001	2.70 (1.97-3.71)	<0.001
eGFR	0.96 (0.95-0.96)	<0.001		
BMI				
<30	Ref			
≥30	1.18 (0.78-1.80)	0.426		
Albumin	0.91 (0.88-0.94)	<0.001	0.88 (0.85-0.92)	<0.001
HEI-2020	1.01 (1.00-1.02)	0.008		

Note: PIR, poverty income ratio; CVD, cardiovascular diseases; eGFR, estimated glomerular filtration rate; BMI, body mass index; HEI-2020, healthy eating index-2020; HR, hazard ratio; Ref, reference.
